# Surgical Club of South West England

**Published:** 1990-06

**Authors:** 


### Surgical Club of South West England
### Meeting in Taunton, Spring 1989

### "DO YOU REALLY UNDERSTAND DIATHERMY"
**G. N. Lumb**, Taunton

Understanding of the method by which diathermy produces cutting, coagulation and haemostasis, is essential for surgeons of all kinds. The key lies in the word "POWER" and "Safety" is the watchword. All diathermy functions depend on power and it is only variations of this quantitatively and qualitatively that changes them from coagulation to cutting.

A simple demonstration of power using a couple of plate electrodes of equal size in series connection with a 60W 240V electric bulb was carried out using the demonstrators arm for the current path. The standard setting of the machine for urological coagulation (under fluid) will illuminate the bulb very adequately.

Direction of the power by safe application of the plate electrode in such a way as to avoid traversing the cardiac axes and nodes is mandatory. Ideal sites are, for head and neck surgery on the right upper arm or behind the right shoulder blade, and for the abdomen and legs the right thigh or buttock, making sure there is good even contact.

Understanding the needs of organs supplied by a single vessel or arteries that are end arteries, leads to the logical step of bi-polar diathermy. Monopolar diathermy even if used with a saline pad is not a complete guarantee that thrombosis of the artery of supply will not occur and consequent necrosis of part or whole of the organ. For circumcision and operations on the testis bipolar diathermy is the only acceptable medico-legal way.

Safety depends on good maintenance of the machines, careful use and adherence to a formal safety code.

### MEDICAL DATA INDEX (MDI)— REGIONAL AUDIT
**S. M. Jones**, Taunton

The assessment of the results of treatment is an integral component of good surgical practice. Most surgeons achieve this function by the regular post-operative follow up of patients and some record their cases in a notebook or card index system.

The availability of desk-top microcomputers has encouraged some clinicians to undertake a more systematic recording of their work and this enthusiasm has been reinforced by the Government White Paper and guidance by the Royal Colleges.

In January 1989 the General Surgeons in Taunton began a pilot study of the SWRHA's Medical Data Index clinical audit system, using software written by the Regional Computer Centre. This system is unique in that it runs on the mainframe computer, accessed via a microcomputer acting as a terminal.

Collected on record cards completed by doctors, the data is entered into the system by medical secretaries and the information stored on the same mainframe computer as the hospital Patient Administration System (PAS). This allows access with the appropriate password via any terminal in the district and direct transfer of data from the PAS avoiding duplication.

Interest has been shown by surgeons in most centres in the South West and the hope is that adoption of this system Region-wide will enable us to collaborate in the establishment of a Regional database for consultant led surgical audit.

### RESTORATIVE PROCTO-COLECTOMY— ILEAL POUCH SYMPOSIUM
**Charles Collins**, Taunton
**Hamish Thomson**, Gloucester
**David Bartolo**, Bristol

Charles Collins presented his experience of six Ileal Pouch operations, indicating the generally good functional results which followed the procedure. One of his patients attended to give a personal account of her symptoms before and after the operation. The importance of the patient's understanding and positive attitude to the procedure were stressed. The various techniques of construction of the ileal pouch were discussed from the original Park's 'S' pouch to the fully stapled 'J' pouch, and the one favoured by surgeons in the South West: the 'W' pouch, practised most frequently by Nicholls of St. Mark's. In the Taunton series the 'W' pouch is anastomosed to the anal canal, using the circular stapling gun.

Mr. David Bartolo confirmed the good functional results and described his experience with 38 patients on whom he had carried out a pouch operation which encompassed a much wider age range. He discussed some of the problems and physiological measurements made of anal function before and after the procedure.

Hamish Thomson commented on his form of case selection and all agreed that restorative procto-colectomy was the treatment of choice for the ulcerative colitic patient not responding to medical treatment. It was, however, contra-indicated in Crohn's disease.

### MAGNETIC RESONANCE IMAGING IN GENERAL SURGERY
**P. M. Cavanagh**, Taunton

Nuclear magnetic resonance imaging (MRI and NMR) has developed rapidly in the past five years to become the imaging modality of choice in most investigations in the central nervous system and spine. More recently still it has established a firm place in the investigation of joint pathology in musculo-skeletal tumours and avascular necrosis.

However, as with CT the application in the chest and abdomen have lagged behind. The advantages of MRI over CT include better contrast resolution of soft tissues, imaging in any plane (not just axially), and the lack of ionising radiation. One of the prices to pay is extended scan times and this obviously leads to movement artefacts from respiration, bowel, the cardiovascular system as well as the patient's voluntary movements.

Such movement artefact is not a problem in the breast and also the pelvis. MRI has, however, been a disappointment in the breast but the pelvis is a different story. NMR has been shown to be as accurate as CT if not more so in staging prostatic, bladder and gynaecological neoplasms.

With the liver, movement artefact is more of a problem but newer sequences have overcome much of this. Although CT is at present the mainstay of the investigation of liver metastases, it only has a sensitivity of 73–74%. Comparison with MRI shows that MRI has a similar sensitivity without intravenous contrast. MRI also has a greater specificity for benign lesions such as haemangioma and cyst.

Thus the indications for MRI in the liver are:

1. Solitary tumours of unknown aetiology
2. Primary tumour if surgery is considered
3. Negative or equivocal CT with strong suspicion of metastases.

Within the rest of the abdomen, although there are numerous publications on the applications of MRI, CT and

Ultrasound remain the primary imaging tests with MRI considered in problem cases.

The applications in the CNS and musculo-skeletal system are on occasions of value to the general surgeon. For instance MRI is superior to other techniques in looking for marrow replacement by metastases, in transplant patients with suspected avascular necrosis, and in investigated neurological causes of bladder disfunction.

On the horizon are developments in contrast media which should increase accuracy in a number of established areas and hopefully will increase its use in other situations. In addition, new vascular sequences are already producing images comparable to early D.V.I. without the introduction of contrast agents.

In conclusion, MRI is the most rapidly developing imaging technique and it is likely it will soon compete with established techniques in much of the general surgeons work.

### NEW APPROACHES TO THE TREATMENT OF MALIGNANT DISEASE

**S. A. N. Johnson**, Taunton

Recombinant DNA technology has made available an increasing number of molecularly cloned and purified growth factors with a wide range of possible clincial uses. Erythropoietin is successful in correcting the anaemia of end-stage renal failure and the corresponding molecule active in stimulating neutrophil production, G-CSF has been successfully used to shorten the duration of neutropenia after cytotoxic treatment for malignant disease.

The interferons have undergone extensive clinical trials in a wide range of malignant diseases. The alpha interferons appear to show the best range of activity and responses in Hairy Cell Leukaemia and Chronic Myeloid Leukaemia are often dramatic; activity in an adjuvant setting is also seen in myeloma, Kaposi's sarcoma, endocrine pancreatic tumours, melanoma and renal cell carcinoma.

The most active area of research currently is into Interleukins which are produced by helper T lymphocytes and of these agents the most promising anti-tumour responses have been achieved with Interleukin 2. Direct infusions of IL-2 have been combined with the preparation of specifically stimulated lymphocytes such as lymphokine-activated killer cells (LAK) or tumour infiltrating lymphocytes (TIL). Activity against renal cell carcinomas and melanomas has been demonstrated, however, complete remissions are few and toxicity is high with fever, hypotension, fluid retention and renal impairment.

BRONCHUD, M. H. and DEXTER, T. M. (1989) Clinical use of growth factors. *Brit Med. Bull.* **45**, 590–599.

### THERAPEUTIC BILIARY ENDOSCOPY

**I. Eyre-Brook**, Taunton

The development of endoscopic retrograde cholangio pancreatography has allowed us to treat many patients with biliary stones or malignant bile duct obstruction without recourse to surgery. Endoscopic sphincterotomy (ES) with stone extraction is now well established as the treatment of choice for bile duct stones after cholecyastectomy. Many elderly patients with symptomless gallbladder stones are now undergoing ES as the definitive treatment for their bile duct stones; subsequently cholecystectomy has proved necessary in approximately 6% of patients followed for 1–2 years. Stones too large to be extracted endoscopically may be fragmented by a variety of techniques currently under assessment, including mechanical crushing, chemical dissolution or shock wave lithotripsy. The use of endoscopically inserted small pig tail stents can prevent stone impaction at the ampulla and allows many patients with large bile duct stones to remain symptomless.

Endoscopic insertion of larger diameter straight stents can relieve jaundice in those with bile duct obstruction from incurable carcinoma of the pancreas or bile ducts. Stents may become blocked and occasionally need replacement. Controlled trials have demonstrated that endoscopic stenting is as effective as bypass surgery in relieving jaundice from carcinoma of the pancreas and involves a shorter initial hospital stay. Stents may be difficult to insert but with the help of the interventional radiologists and the use of a combined percutaneous and endoscopic approach stenting can usually be achieved.

Surgery still has an important part to play in the management of biliary tract obstruction. We need more clinical studies to establish the correct balance between surgery and endoscopic therapy.

### VIDEO-ENDOSCOPY

**P. J. O'Boyle**, Taunton

Advances in optical and electronic technology in the early 1980's permitted the introduction of a microvideo camera to the Urologist's armamentarium. The technique of videoprostatectomy or operating from the image on a video monitor rather than by peering down a telescope, was pioneered in Taunton and has subsequently become standard practice in many of the leading urological centres throughout the world. The video reviews the development of the microvideo camera and its application for a wide range of urological operating techniques. Initially the use of the camera in transurethral prostatic surgery was introduced with caution, but subsequently it has become apparent that a satisfactory image in terms of colour and resolution, can now permit the attachment of the microvideo camera to virtually any rigid optical instrument. Examples of resection of bladder tumour, injection of Teflon, ureterorenoscopy and percutaneous stone extraction demonstrated the versatility of the microvideo operating system in the busy District General Hospital Department of Urology.

### ECTOPIC THYMIC CYSTS

**A. E. Adam**, Taunton

"A wandering thymus I
A thing of branchial pouches
Of painless cysts, not ouches
A curious ectopy . . . " (after Nanki-Poo)

The thymus gland starts life as paired buddings off the third branchial pouches. Usually they sink without trace down the neck to their mediastinal destination. Ectopic tissue, however, may persist anywhere along the migration path and present surgically as a cystic neck swelling in a youngster, or less often as a solid tumour at parathyroid surgery. Ectopic thymoma is very rare.

A personal collection of 15 ectopic thymic cysts added to sporadic cases in the literature brings together at least 72 patients in 41 reports. The "average" patient is a 13 year old boy with a painless neck swelling, often fluctuating in size and present for eight months. There is a left sided and a male dominance (both 2:1).

These cysts are almost impossible to diagnose preoperatively and are mistaken for thyroid or thyroglossal cysts, branchial cleft cysts and cystic hygromas. When the diagnosis is suggested preoperatively, the presence of a mediastinal thymus should be established by CT scan to avoid an inadvertent total thymectomy.

Over half the specimens have a vestigial connection with the mediastinum and may be sausage-like (up to 15 cm), requiring extensive dissection. Histologically they pose no problems, except in frozen sections; parathyroid or thyroid tissue is occasionally admixed. The microscopic features suggest a degenerative change occurring at the time of thymic involution.

